# Parasitological, serological and molecular survey of camel trypanosomiasis in Somalia

**DOI:** 10.1186/s13071-019-3853-5

**Published:** 2019-12-21

**Authors:** Ahmed A. Hassan-Kadle, Abdalla M. Ibrahim, Hamisi S. Nyingilili, Abdulkarim A. Yusuf, Thállitha S. W. J. Vieira, Rafael F. C. Vieira

**Affiliations:** 1 Abrar Research and Training Centre, Abrar University, Mogadishu, Somalia; 2 Department of Veterinary Medicine, Universidade Federal do Paraná, Curitiba, PR Brazil; 3 Vector and Vector Borne Diseases Institute, Tanga, Tanzania; 4 Global One Health Initiative (GOHi), The Ohio State University, Columbus, OH USA

**Keywords:** CATT/*T. evansi*, Dromedary, ITS1-PCR, *Trypanosoma evansi*, *Trypanosoma simiae*

## Abstract

**Background:**

Camel trypanosomiasis or surra is of great concern in Somalia, since the country possesses the largest one-humped camel (*Camelus dromedarius*) population in the world. Civil war in Somalia has resulted in the destruction of educational, research, economic and social structures, making the country scores very low for most humanitarian indicators. Previous studies on detection of *Trypanosoma* species in Somali camels have only been performed during the 1990s using standard trypanosome detection methods (STDM). Considering the lack of state-of-the-art knowledge on camel trypanosomiasis in Somalia, the present study aimed to assess the prevalence of *Trypanosoma* spp. in three districts of Somalia.

**Methods:**

A total of 182 blood samples from *C. dromedarius* from nomadic and dairy farms were evaluated using STDM, serological (CATT/*T. evansi*) and molecular (ITS1-PCR) methods.

**Results:**

All samples were negative for *Trypanosoma* spp. by STDM. A total of 125/182 (68.7%, 95% CI: 61.4–75.3%) camels were seropositive for *T. evansi* by CATT/*T. evansi*. Camels reared in nomadic system were more likely to be seropositive for *T. evansi* than those under dairy production system (OR: 5.6, 95% CI: 2.1–15.2, *P* = 0.0001). Five out of 182 (2.7%, 95% CI: 0.9–6.3%) camels tested positive for *Trypanosoma* sp. by ITS1-PCR. Sequencing of the ITS1 region of the *Trypanosoma* species detected herein revealed that camels were infected with *T. evansi* and *T. simiae*.

**Conclusions:**

*Trypanosoma evansi* is highly prevalent in camels from the Banadir region of Somalia, particularly in nomadic herds. To our knowledge, this is the first study to confirm infections with *T. evansi* and *T. simiae* in Somali camels through DNA sequencing. Our data highlight the need for implementation of adequate control measures aiming to reduce the impact on camel production in the country.

## Background

Trypanosomiasis are vector-borne diseases (VBD) that causes noticeable economic losses [1–3] and affects the development of both livestock and human health in Africa [4]. In Somalia, camel trypanosomiasis or surra is of great concern since the country possesses the largest one-humped camel (*Camelus dromedarius*) population in the world, estimated at nearly 8,000,000 heads [5–7]. The economic importance of camels for Somalia is due to their role as a food source, as currency, as a means of transporting milk and water as well as an indicator of social issues [7]. Camels are uniquely adapted to survive and produce under extreme arid and semi-arid conditions of Somalia [7], with the majority of animals kept by nomadic pastoralists in the country [6, 7].

Civil war in Somalia has resulted in the destruction of educational, research, economic and social structures, making the country score very low for most humanitarian indicators [8]. Currently, Somali communities and their livestock are experiencing a famine and suffering from preventable diseases, due to geographical and political isolation and lack of state-of-the-art knowledge. Recently, after some security and political settlement in the country, camels are kept around urban areas as a semi-intensive dairy farming system.

Worldwide, camels may be affected by tsetse-transmitted *Trypanosoma* species, including *T. simiae*, *T. brucei*, *T. congolense* and *T. vivax* [9, 10]. In Somalia, previous studies on detection of *Trypanosoma* species were performed during the 1990s and reported *T. evansi* prevalence rates ranging from 1.7% to 56.4% in camels [7, 11]. Additionally, *T. simiae* [9], *T. congolense* and *T. brucei* have also been detected in Somali camels by standard trypanosome detection methods (STDM) [11].

Clinical signs of trypanosomiasis may be absent in camels, and thus, laboratory diagnosis should be carried out for confirmation of infection. Several methods with varying degrees of sensitivity and specificity may be used for the diagnosis of trypanosomiasis. Standard trypanosome detection methods, such as microscopical examination of fresh or stained blood-smears, has been historically used in the identification of *Trypanosoma* spp. Unfortunately, this technique lacks sensitivity and specificity. A serological assay, the card agglutination test for *T. evansi* (CATT/*T. evansi*) is a rapid diagnostic test and currently recommended by the World Organization for Animal Health [2, 12]. Additionally, molecular analysis targeting the internal transcribed spacer 1 (ITS1) region provides multi-species-specific detection of trypanosomes in a single PCR [13], and has been used in epidemiological studies.

Although the National Tsetse and Trypanosomiasis Control Project (NTTCP) was established in the 1980s in Somalia, no control measures have been implemented to date. Accordingly, considering the lack of state-of-the-art knowledge on camel trypanosomiasis in Somalia, the present study aimed to assess the prevalence of camel trypanosomiasis in three districts of Somalia using STDM, serological (CATT/*T. evansi*) and molecular (ITS1-PCR) methods.

## Methods

### Study area

Banadir region is one of the eighteen regions of the Federal Republic of Somalia. The region itself is coextensive with Mogadishu city, the capital of the country. It consists of 17 districts, and three of them were included in this study: Kahda (2° 4′ 4.17″ N, 45° 14′ 16.16″ E), Daynile (2° 4′ 24.61″ N 45° 16′ 48″ E) and Yaqshid (2° 4′ 3.97″ N, 45° 21′ 35.9″ E). These districts are the main camel rearing areas in the investigated region.

### Study animals and blood sampling

From December 2015 to March 2016, which represents the dry season in Somalia, a total of 182 *C. dromedarius* (176 females and 6 males) ≥ 2 years-old from nomadic (*n* = 49) and dairy (*n* = 133) farms in the Kahda (*n* = 72), Daynile (*n* = 87) and Yaqshid (*n* = 23) districts were randomly evaluated. Blood samples were collected by jugular venipuncture. Three millilitres were placed into tubes without anticoagulant and kept at room temperature (25 °C) until visible clot retraction; the samples were then centrifuged at 1500× *g* for 5 min, serum separated and kept at − 20 °C for serological studies. One ml was placed into EDTA tubes for packed cell volume (PCV) measurement, microscopical detection of trypanosomes and preparation of blood spots on filter paper (Whatman no.4, Whatman, Springfield Mill, United Kingdom) for PCR analysis. A PCV of 0.26 l/l or less was used as an indicator of anaemia [14].

### Parasitological diagnosis of *Trypanosoma* spp.

All camel blood samples were evaluated for the presence of *Trypanosoma* spp. by STDM. Briefly, a drop of fresh whole blood (after gentle mixing) was placed on a clean microscope slide, covered with coverslip and examined for the motile parasites, as previously described [15]. Giemsa-stained thin blood and buffy coat smears were also examined for the presence of *Trypanosoma* spp., as described elsewhere [2].

### Detection of *T. evansi* antibodies by card agglutination test (CATT/*T. evansi*)

Camel serum samples were tested for the presence of *T. evansi* antibodies using the card agglutination test (CATT/*T. evansi*) [16], according to the manufacturer’s instructions (Institute of Tropical Medicine, Antwerp, Belgium).

### DNA extraction and PCR for *Trypanosoma* spp.

Genomic DNA was extracted from all 182 dried blood spots by Chelex-100 (Sigma-Aldrich, St. Louis, USA), as previously described [17]. The DNA samples were evaluated by a PCR assay targeting the ITS1 region of *Trypanosoma* species using previously described primers [18]. The PCR amplifications were performed in a total reaction volume of 25 μl containing 0.5 μl of 10 pM of each primer, 12.5 μl of 2× master mix (New England BioLabs, Ipswich, MA, USA), 9.5 μl of PCR water and 2 μl of each DNA template. PCR amplifications were performed with a thermal cycler (GeneAmp^®^ PCR System 9700, Applied Biosystems^®^, Foster City, CA, USA). The amplification conditions used included an initial denaturation at 94 °C for 30 s, followed by 30 cycles of 94 °C for 30 s, 58 °C for 40 s, 68 °C for 1 min, with a final extension step at 68 °C for 5 min and cooling at 4 °C. Nuclease-free water and a *T. evansi*-positive sample were used as negative and positive control, respectively, in all PCR runs. The amplified PCR products were analysed by electrophoresis in a 1.5% agarose gel at 100–120 V for 60 min. Quick Loading 100 bp DNA ladder (New England BioLabs) was included on each gel, stained with ethidium bromide, and finally visualized under ultraviolet (UV) illuminator (UVITEC™, Cambridge, UK).

### Sequencing and phylogenetic analysis

Amplicons (~ 400 bp) obtained from two *Trypanosoma*-positive samples were sequenced in both directions by the Sanger method and were assembled using Geneious Prime^®^ 2019.1. Consensus sequences were subjected to BLASTn analysis [19] for determining the identity with the sequences deposited in the GenBank database.

The *Trypanosoma* ITS1 region sequences (GenBank: MH885470, MH885471) were aligned with sequences from GenBank using ClustalW [20] and alignments were improved using GUIDANCE2 [21]. The best-fit model of nucleotide substitution was determined using jModeltest v.2.1.10 [22] and was set as F81+G in the maximum likelihood (ML) phylogenetic estimation on the CIPRES Science Gateway [23], including 1000 bootstrap replicates. The resulting tree was visualized using FigTree software version 1.4.3 [24] and the final layout was rendered using Inkscape version 0.92.3 [25].

### Data management and analysis

The PCV data were not normally distributed (Shapiro–Wilk normality test, *W* = 0.98, *P* = 0.018). Therefore, a non-parametric Mann–Whitney test was used to compare the PCV concentration between *Trypanosoma*-infected and non-infected camels. Either Chi-square or Fisher’s exact test was used to assess association of the individual variables (district and production system) with *Trypanosoma* spp. infection. Odds ratio (OR), 95% confidence intervals (95% CI) and *P*-values were calculated, and results were considered significant when *P* < 0.05. Data were compiled and analysed by Statistical Package for Social Sciences (SPSS) version 25 (IBM Corp., Armonk, NY, USA).

## Results

All samples were negative for *Trypanosoma* spp. by STDM. A total of 125/182 (68.7%, 95% CI: 61.4–75.3%) camels were seropositive for *T. evansi* by CATT/*T. evansi*. Camels reared in the nomadic system were more likely to be seropositive to *T. evansi* than those reared under the dairy production system (OR: 5.6, 95% CI: 2.1–15.2, *P* = 0.0001). No associations between seropositivity for *T. evansi* and the three districts of Somalia evaluated were found (*P* > 0.05). The prevalence of *Trypanosoma* sp. for each variable evaluated is summarized in Table 1.

**Table 1:** Prevalence of camel trypanosomiasis within each variable studied

Variable	CATT*/T. evansi*				ITS1-PCR			
	+/*n*	Prevalence (%) (95% CI)	*P*-value	OR (95% CI)	+/*n*	Prevalence (%) (95% CI)	*P*-value	OR (95% CI)
Production system								
Nomadic	44/49	89.8 (77.8–96.6)	0.0001 (*χ*^2^ = 13.9)	5.6 (2.1–15.2)	2/49	4.1 (0.5–14)	0.408 (*χ*^2^ = 0.45)	1.8 (0.3–11.4)
Dairy	81/133	60.9 (52.1–69.2)			3/133	2.3 (0.5–6.5)		
District								
Daynile	62/87	71.3 (60.6–80.5)	0.242 (*χ*^2^ = 1.38)	1.5 (0.8–2.9)	2/87	2.3 (0.3–8.1)	0.502 (*χ*^2^ = 0.45)	0.5 (0.09–3.33)
Yaqshid	18/23	78.3 (56.3–92.5)	0.164 (*χ*^2^ = 1.94)	2.2 (0.7–6.5)	0/23	0.0 (0.0–14.8)	0.989 (*χ*^2^ = 0.32)	0.0
Kahda	45/72	62.5 (50.3–73.6)			3/72	4.2 (0.9–8.8)		

*Abbreviations*: +, number of positive animals; *n*, number of samples; 95% CI, 95% confidence interval; OR, odds ratio

Five out of 182 (2.7%, 95% CI: 0.9–6.3%) camels tested positive for *Trypanosoma* spp. by ITS1-PCR. Concordant results for *Trypanosoma* spp. presence determined by CATT/*T. evansi* and ITS1-PCR were found in three of 182 camels (1.6%, 95% CI: 0.3–4.7%).

The mean PCV concentration for camels was 0.27 l/l. A total of 61/182 (33.5%, 95% CI: 26.7–40.9%) camels were anaemic. No statistical difference (*U* = 2944, *Z* = − 1.89, *P* = 0.059) was found in mean PCV between *Trypanosoma*-seropositive (0.27 l/l) and *Trypanosoma-*seronegative camels (0.28 l/l). Association between *Trypanosoma* infection and anaemia was not found (*χ*^2^ = 1.93, *df*  = 1, *P* = 0.165).

**Fig. 1:**
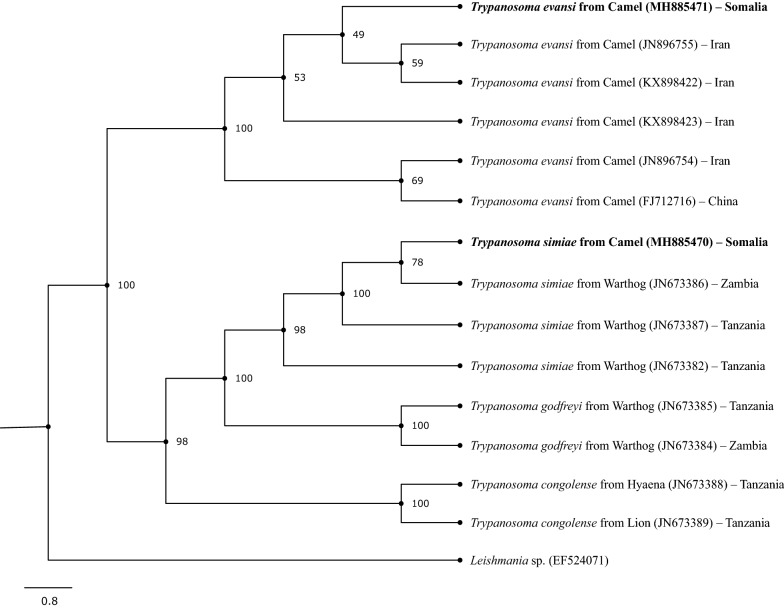
Phylogenetic relationships of *Trypanosoma* spp. evaluated in camels based on ITS1 region sequence with selected sequences from GenBank (Accession numbers in the figure). The ITS1 tree was rooted with *Leishmania* sp. (GenBank: EF524071)

Five *Trypanosoma*-positive samples were sequenced; however, only two sequences yielded consistent data. One *Trypanosoma*-positive sample (GenBank: MH885471) sequenced showed 99.78% (460/461 bp) identity to *T. evansi* ITS1 region sequences detected in camels from Iran (GenBank: KX898422, KX898423). The other sequence obtained (GenBank: MH885470) showed 99.25% (398/401 bp) and 98% (393/401 bp) identity with *T. simiae* ITS1 region sequence from warthogs (*Phacochoerus africanus*) of Tanzania and Zambia, respectively (GenBank: JN673387 and JN673386). The phylogenetic tree based on sequences of the ITS1 region indicated that *T. evansi* obtained herein was closely related to *T. evansi* detected in camels from Iran, whereas *T. simiae* detected in the present study was closely related to *T. simiae* detected in warthog from Zambia (Fig. 1).

## Discussion

To the author’s knowledge, this is the first study to combine STDM, serological and molecular detection of *Trypanosoma* and assess these results for potential associations with epidemiological data collected from camels in Somalia. Herein, overall 69.8% camels from the Banadir region of Somalia were positive for *Trypanosoma* spp. Interestingly, *Trypanosoma* prevalence found herein was higher than previous studies performed in camels from Somalia which have shown prevalence rates ranging from 1.7% to 56.4% by STDM [7, 11] and complement fixation test [7]. Differences among the prevalence of *Trypanosoma* may be explained by the camel population and management, diagnostic test used, and tsetse seasonal dynamics (rainy *vs* dry season).

Sequencing of the ITS1 region of the *Trypanosoma* species detected herein revealed that camels were infected with *T. evansi* and *T. simiae* (Fig. 1). Although previous studies in Somalia have reported *T. evansi*, *T. simiae*, *T. brucei* and *T. congolense* infecting camels by STDM, the present study is the first to confirm infections with *Trypanosoma* spp. in camels in this country by DNA sequencing.

In the present study, all animals were negative for *Trypanosoma* by STDM, while the molecular and serological prevalence were 2.7% and 68.7%, respectively. Combining CATT/*T. evansi* and ITS1-PCR has increased the prevalence of *Trypanosoma* to 69.8%, corroborating with previous studies suggesting that improved sensitivity and specificity for detection of VBD pathogens can be achieved using different diagnostic methods [26]. The difference between the ratio of ITS1-PCR and parasitological method found herein may be explained by low parasitaemia which is typical for the chronic phase of infection [27]; this is also supported by the high seroprevalence observed indicating that a large proportion of camels are exposed to the parasite.

Herein, the *T. evansi* seroprevalence rate was significantly higher in nomadic camels as compared to dairy farm camels (*P* = 0.0001). Previous studies on Somali camels have shown that animals living in riverine zones of the country were more likely to acquire infection by *Trypanosoma* species than those living in inland zones [7]. The camels’ role in the subsistence sector is not primarily for supply of meat and money, but mainly for provision of milk [7]. Considering that nomadic herders usually depend on traditional ethno-veterinary remedies to treat and prevent diseases in their camels [28, 29], the high *T. evansi* seroprevalence found herein highlights the need for implementation of adequate control measures aiming to reduce the impact of trypanosomes on camel production in Somalia. On the other hand, dairy animals evaluated in the present study were frequently examined by veterinary practitioners and treated with Suramin (data not shown), which may explain the low seroprevalence found.

## Conclusions

*Trypanosoma evansi* is highly prevalent in camels from the Banadir region of Somalia, particularly in nomadic herds. To our knowledge, this is the first study to confirm infections with *T. evansi* and *T. simiae* in Somali camels by DNA sequencing. Our data highlight the need for implementation of adequate control measures aiming to reduce the impact of trypanosomes on camel production in the country, which possesses the largest one humped camel population in the world.

## Data Availability

Data supporting the conclusions of this article are provided within the article. Sequences were submitted to the GenBank database under the Accession numbers MH885470 and MH885471 for *Trypanosoma* ITS1 region of *T. simiae* and *T. evansi*, respectively.

## Abbreviations

ARTC: Abrar Research and Training Centre
AU: Abrar University
BLAST: basic local alignment search tool
CATT: card agglutination test for trypanosomiasis
CI: confidence interval
DNA: deoxyribonucleic acid
GOHi: Global One Health Initiative
ITS: internal transcribed spacer
ML: maximum likelihood
NTTCP: National Tsetse and Trypanosomiasis Control Project
OR: odds ratio
PCR: polymerase chain reaction
PCV: packed cell volume
SPSS: statistical package for social sciences
STDM: standard trypanosome detection methods
UV: ultraviolet
VBD: vector-borne diseases
VVBDI: Vector and Vector Borne Diseases Institute
